# Comparison of laparoscopic, robotic, and open retroperitoneal lymph node dissection for non-seminomatous germ cell tumor: a single-center retrospective cohort study

**DOI:** 10.1007/s00345-023-04459-z

**Published:** 2023-06-19

**Authors:** Juntao Lin, Zhenghui Hu, Shihan Huang, Bohua Shen, Shuo Wang, Jianjun Yu, Ping Wang, Xiaodong Jin

**Affiliations:** 1 Department of Urology, The First Affiliated Hospital, Zhejiang University School of Medicine, Hangzhou, 310003 Zhejiang People’s Republic of China; 2 Department of Urology, Zhejiang Provincial Hospital of Chinese Medicine, Zhejiang Chinese Medical University, Hangzhou, 310000 Zhejiang People’s Republic of China

**Keywords:** Laparoscopic surgery, Open surgery, Robotic Surgery, Testicular cancer, Retroperitoneal lymph node dissection

## Abstract

**Purpose:**

To compare the perioperative outcomes of L-RPLND, R-RPLND and O-RPLND, and determine which one can be the mainstream option.

**Methods:**

We retrospectively reviewed medical records of 47 patients undergoing primary RPLND by three different surgical techniques for stage I–II NSGCT between July 2011 and April 2022 at our center. Standard open and laparoscopic RPLND was performed with usual equipment, and robotic RPLND was operated with da Vinci Si system.

**Results:**

Forty-seven patients underwent RPLND during 2011–2022, and 26 (55.3%) of them received L-RPLND, 14 (29.8%) were operated with robot, while 7 (14.9%) were performed O-RPLND. The median follow-up was 48.0 months, 48.0 months, and 60.0 months, respectively. The oncological outcomes were comparable among all groups. In L-RPLND group, there were 8 (30.8%) cases of low grade (Clavien I–II) complications, and 3 (11.5%) cases of high-grade (Clavien III–IV) complications. In R-RPLND group, one (7.1%) low-grade complication and four (28.6%) high-grade complications were observed. In O-RPLND group, there were 2 (28.5%) cases of low-grade complications and one case (14.2%) of high-grade one. The operation duration of L-RPLND was the shortest. In O-RPLND group, the number of positive lymph nodes were higher than other two groups. Patients undergoing open surgery had lower (*p* < 0.05) red blood cell count, hemoglobin level, and higher (*p* < 0.05) estimated blood loss, white blood cell count than those receiving either laparoscopic or robotic surgery.

**Conclusion:**

All three surgical techniques are comparable in safety, oncological, andrological, and reproductive outcomes under the circumstance of not using primary chemotherapy. L-RPLND might be the most cost-effective option.

## Introduction

Among all kinds of malignancies, testicular cancer is relatively rare and less lethal. Despite of its rarity and high survival rate, it is the most common solid tumor in male population between 20 and 34 years. Because of their contribution to labor force and fertility [1], an optimal treatment is essential for this disease.

For stage I–IIB testicular non-seminomatous germ cell tumor (NSGCT), retroperitoneal lymph node dissection (RPLND) is the recommended treatment, especially in patients with teratoma, according to guidelines published both in America and Europe [2, 3]. Although both chemotherapy and RPLND are approved for primary treatment of NSGCT, for patients anxious about the potential long-term toxicities of cytotoxic drugs, surgery may be a preferred choice [4]. To date, there are three surgical techniques for RPLND: open RPLND (O-RPLND), which is the standard procedure; laparoscopic RPLND (L-RPLND) and robotic RPLND (R-RPLND) as proper minimally invasive alternatives when access to particular expertise is available [3].

Comparisons between O-RPLND and L-RPLND were performed by several studies after the introduction of laparoscopic technique into this treatment, demonstrating the superiority of L-RPLND in operative time, complication rate, blood loss, hospital stay and life quality over O-RPLND. The economic advantage of O-RPLND was also investigated [5]. With the advancement of robotic surgery for urological diseases such as prostate cancer and renal tumors, the feasibility and safety of R-RPLND were also verified [6]. However, whether it can replace traditional O-RPLND and L-RPLND remains inexplicit, partly due to its unpredictable adverse surgical outcomes reported by Calaway et al. [7]. Meanwhile, the morbidity rate and oncological outcomes between R-RPLND and L-RPLND are also comparable [8]. Most of such comparisons focused on surgical related parameters and oncological results, and some recent studies began to highlight the impacts of these techniques on life quality, especially andrological problems such as anejaculation and infertility [9, 10]. However, comprehensive comparisons of all three modalities conducted in one medical center involving analysis of their impacts on blood tests results have not yet published.

In this study, we performed a single-centered retrospective cohort study in a urological center in eastern China with expertise on all three surgical methods, aiming to compare the comprehensive outcomes including short-term impacts on patients’ blood tests results between L-RPLND, R-RPLND and O-RPLND, and propose an optimal choice for future treatment.

## Patients and methods

### Study design

After exclusion of 2 patients with non-testicular cancer histology and one performing primary chemotherapy, we retrospectively reviewed medical records of 47 patients undergoing primary RPLND by three different surgical techniques for stage I–II NSGCT between July 2011 and April 2022 at the First Affiliated Hospital, Zhejiang University School of Medicine. It was approved by the Clinical Ethics Committee and conducted according to the institution guidelines. All patients included were provided with every available management option such as active surveillance, chemotherapy and RPLND, in accordance with up-to-date guidelines [2].

### Surgical technique and surgeons’ information

For patients undergoing L-RPLND, they were firstly positioned laterally according to the laterality of the lesion. Then a 12-mm periumbilical camera trocar was located after Veress needle insufflation while two additional working trocars were placed in the lower and upper quadrants. After laparoscopic access was ready, the boundary of the right unilateral template was defined by right renal artery in superior direction, bifurcation of the right common iliac artery in inferior direction, the right ureter in lateral direction and pre-aortic nodes in medial direction. As for left unilateral template, the boundary was renal arteries in superior direction, the bifurcation of the common iliac arteries in inferior direction, the left ureter in lateral direction, and the inferior vena cava in medial direction [11]. Sympathetic trunk was paid special attention to when possible. All specimens were collected through entrapment sac and removed from the extended camera port site.

For those receiving R-RPLND, the da Vinci Si system was used with a 12-mm camara port positioned 3–4 cm beneath the umbilicus on the midline, two 8-mm robotic trocars placed in the left lower quadrant and an 8-mm robotic trocar accompanied with a 12-mm one in the right lower quadrant. Despite of different template laterality, port placement remained the same. The boundary of a modified template dissection was the same as laparoscopic surgery. Lymph nodes dissected included the paracaval, interaortocaval and pre-aortic ones for right-sided procedures, while the para-aortic, interaortocaval and pre-caval lymph nodes were dissected for left-sided procedures [12].

The limits of the template of O-RPLND were similar to L-RPLND, and dissection was performed to the medial boundary of the aorta for the right-sided template and the medial boundary of the vena cava for the left-sided template.

The selection of the operating modulation for each patient was depended on the expertise of the patient’s surgeon in charge and the patient’s preference when different choices were available. All surgeries were performed by experienced surgeons. The laparoscopic procedures were conducted by Dr. Xiaodong Jin and Dr. Jianjun Yu. Dr. Jin performed 15 cases of L-RPLND and Dr. Yu conducted the other 11 cases. Both performed more than 2000 various laparoscopic urological surgeries, including prostatectomy, nephrectomy, and cystectomy, during their 24-year careers. The certified da Vinci robotic operation experts in our center, Dr. Shuo Wang and Dr. Ping Wang, completed all 14 R-RPLND cases. The open ones were all performed by Dr. Bohua Shen, a highly skilled urological surgeon performing over 100 open surgeries every year for 30 years.

### Post-operative management

A low-fat diet (≤ 20 g fat/d) for at least 2 weeks was encouraged to prevent chylous ascites. Standard follow-up procedure was conducted in consistent with National Comprehensive Cancer Network guidelines [13] for markers and radiological imaging. Chemotherapy was given to stage II patients.

### Statistical analysis

Statistical analysis was performed using SPSS Statistics^®^ 26 (IBM, USA). Demographic, pathological, pre- and post-operative data were analyzed among L-RPLND group, R-RPLND group, and O-RPLND group. Continuous variables were compared with univariate ANOVA and Mann–Whitney test, while categorical variables with Fisher’ s exact test. Statistical significance in this study was set at *p* < 0.05.

## Results

### Patient cohort

A total of 47 patients underwent RPLND during 2011–2022, and 26 (55.3%) of them received L-RPLND, 14 (29.8%) were operated robotically, while 7 (14.9%) were performed O-RPLND. The mean age of the 46 patients in L-, R-, and O-RPLND groups was 33.3 years, 33.9 years and 32.6 years, respectively. The majority of them (63.8%) were diagnosed as pathological stage I. Post-operative chemotherapy including 16 BEP regimens and one EP regimen. The median follow-up was 48.0 months (IQR 12.0–96.0), 48.0 months (IQR 24.0–60.0), and 60.0 months (21.0–105.0) for patients in L-RPLND group, R-RPLND group, and O-RPLND group (Table 1).

**Table 1:** Demographic and oncological characteristics of all patients

Variable	Combined	L-RPLND	R-RPLND	O-RPLND	*p* value
No	47	26	14	7	
Median (IQR)					
Age, years		32.0 (29.0–38.5)	34.0 (25.0–38.2)	33.0 (22.0–45.0)	0.964
BMI, kg/m^2^		22.6 (21.0–23.7)	22.5 (21.0–25.6)	20.4 (18.5–25.3)	0.579
Following Time (months) *N* (%)		48.0 (12.0–96.0)	48.0 (24.0–60.0)	60.0 (21.0–105.0)	
Primary tumor laterality					0.822
Right	22 (46.8)	12 (46.2)	6 (42.9)	4 (57.1)	
Left	25 (53.2)	14 (53.8)	8 (57.1)	3 (42.9)	
Pathological stage					0.217
IA	28 (59.5)	16 (61.5)	10 (71.4)	2 (28.6)	
IB	2 (4.3)	1 (3.8)	0	1 (14.3)	
IIA	13 (27.6)	8 (30.8)	2 (14.3)	3 (42.9)	
IIB	4 (8.6)	1 (3.8)	2 (14.3)	1 (14.3)	
Histology					
Pure seminoma	0	0	0	0	
Mixed GCT/NSGCT	47 (100.0)	26 (100.0)	14 (100.0)	7 (100.0)	
Teratoma					0.975
Yes	26 (55.3)	14 (53.8)	8 (57.1)	4 (57.1)	
No	21 (44.7)	12 (46.2)	6 (42.9)	3 (42.9)	
Post-operative chemotherapy					
BEP	16	9 (34.6)	4 (28.6)	3 (42.9)	
EP	1	0	0	1 (14.3)	
IGCCC classification					0.259
Good	40 (85.1)	23 (88.5)	10 (71.4)	7 (100.0)	
Intermediate	7 (14.9)	3 (11.5)	4 (28.6)	0	
Poor	0	0	0	0	

### Pre- and post-operative outcomes

When conducting univariate analysis, there were no differences of age and BMI between all three groups. Other baseline, tumor and pre-operative characteristics, including primary tumor laterality, pathological stage, whether teratoma existed, the IGCCC level, red blood cell count, hemoglobin level, white blood cell count, platelet count, albumin level, and total bilirubin level were also comparable. We also recorded the IIEF-5 score of all patients. Four patients (15.4%) in L-RPLND group, 2 (14.3%) in R-RPLND group, and 2 (28.6%) in O-RPLND group were described as lower than 25. The results were also comparable among all groups (Table 2).

**Table 2:** Pre-operative blood tests results and sexual function assessment

	L-RPLND	R-RPLND	O-RPLND	*p* value
RBC (× 10^9^/L)	5.03 ± 0.37	4.91 ± 0.36	4.72 ± 0.65	0.210
WBC (× 10^6^/L)	7.42 ± 2.56	6.80 ± 1.50	5.67 ± 1.38	0.165
Hb (g/L)	152.5 ± 10.3	151.3 ± 14.4	143.9 ± 21.5	0.337
PLT (× 10^9^/L)	225.0 ± 61.0	245.4 ± 43.0	238.0 ± 71.0	0.570
Albumin (g/L)	44.9 ± 4.2	46.1 ± 4.0	45.5 ± 4.3	0.641
Total bilirubin (μmol/L)	12.9 ± 6.0	11.2 ± 5.7	10.6 ± 4.6	0.517
Creatinine (μmol/L)	74 ± 9	85 ± 11*	87 ± 12*	0.001
IIEF-5 < 25, *n* (%)	4 (15.4)	2 (14.3)	2 (28.6)	0.746

*p < 0.05 when compared with L-RPLND

The median operative duration was 162 min (IQR 149–192) in L-RPLND group, statistically lower than 218 min (IQR 166.5–255) in R-RPLND group (*p* < 0.05). In O-RPLND group, that was 209 min (IQR 164–253). The median number of lymph nodes retrieved was 12.5 (IQR 8.5–16), 16 (IQR 4.5–26), and 13 (IQR 7–19), the median length of stay was 12 days (IQR 9–15), 11.5 days (IQR 7–15), and 14 days (IQR 12–27), and the median total hospital expenses were 23,011 RMB (IQR 17389–27,293), 77,866 RMB (IQR 70494–83,765), and 27,214 RMB (IQR 16030–36,851) in each group, respectively (Table 3).

**Table 3:** Comparison of intraoperative and post-operative outcomes between L-RPLND, R-RPLND and O-RPLND

	L-RPLND	R-RPLND	O-RPLND	*p* value
Intraoperative outcomes, median (IQR)				
Operative duration (min)	162 (149–192)	218 (166–255)*	209 (164–253)	0.192
Estimated blood loss (ml)	50 (20–100)	50 (40–60)	150 (150–300)*	0.000
Number of lymph nodes retrieved (*n*)	12.5 (8.5–16)	16 (4.5–26)	13 (7–19)	0.648
Number of positive lymph nodes	0 (0–2)	0 (0–2)	0 (0–6)*	0.029
Post-operative complication, *n* (%)				0.383
Clavien grade I–II	8 (30.8)	1 (7.1)	2 (28.5)	
Clavien grade III–IV	3 (11.5)	4 (28.6)	1 (14.2)	
Antegrade ejaculation	26 (100)	14 (100)	6 (85.7)	0.149
Erectile dysfunction	7 (26.9)	4 (28.6)	2 (28.6)	1.000
Post-operative blood tests, mean ± SD				
RBC (× 10^9^/L)	4.67 ± 0.39	4.56 ± 0.44	4.22 ± 0.65	0.072
WBC (× 10^6^/L)	11.68 ± 3.47	11.80 ± 2.63	16.16 ± 5.77*	0.019
Hb (g/L)	142.7 ± 11.1	138.4 ± 14.9	129.9 ± 18.0*	0.091
PLT (× 10^9^/L)	201.4 ± 65.0	202.1 ± 43.1	187.9 ± 50.9	0.842
Albumin (g/L)	36.9 ± 4.6	35.9 ± 3.2	36.4 ± 4.3	0.779
Total bilirubin (μmol/L)	15.0 ± 6.1	17.7 ± 7.0	13.7 ± 7.7	0.343
Creatinine (μmol/L)	68 ± 11	75 ± 23	71 ± 11	0.112
Medical burdens, median (IQR)/*n* (%)				
Length of stay (days)	12 (9–15)	11.5 (7–15)	14 (12–27)	0.247
Hospital expenses (RMB)	23,011 (17,389–27,293)	77,866 (70,494–83,765)*	27,214 (16,030–36,851)	0.000
Recurrence	1 (3.8)	2 (14.2)	2 (28.5)	0.079

*IQR* interquartile range, *BMI* body mass index, *GCT/NSGCT* germ cell tumor/nonseminomatous germ cell tumor, *BEP* bleomycin, etoposide, and cisplatin, *EP* etoposide and cisplatin, *IGCCC* International Germ Cell Consensus Classification, *RPLND* retroperitoneal lymph node dissection, *RBC* red blood cell, *WBC* white blood cell, *Hb* hemoglobin, *PLT* platelet, *IIEF-5* 5-item version of the International Index of Erectile Function, *SD* standard deviation

*p < 0.05 when compared with L-RPLND

Analysis of post-operative variables suggested that no differences were found between all groups regarding number of lymph nodes retrieved, platelet count, albumin level, total bilirubin level, creatinine level, hospital length of stay, and recurrence rate. The operation duration of L-RPLND was the shortest. In O-RPLND group, the number of positive lymph nodes were slightly higher than other two groups. Patients undergoing open surgery had statistically lower (*p* < 0.05) red blood cell count, hemoglobin level, and higher (*p* < 0.05) estimated blood loss, white blood cell count than those receiving either laparoscopic or robotic surgery. Robotic surgery was the most expensive method in comparison with other surgical techniques, and there was no statistical difference between laparoscopic surgery and open one. Significant decrement of red blood cell count, hemoglobin and albumin levels, and increment of white blood cell count and total bilirubin level with statistical difference were observed one day after the procedure compared with pre-operative results.

### Complications

In L-RPLND group, there were 8 (30.8%) cases of low grade (Clavien I–II) complications, including one case of pelvic fluid and one episode of scrotal edema requiring diuresis, and 3 (11.5%) cases of high-grade (Clavien III–IV) complications, all of which were chylous ascites managed with percutaneous drainage and low-fat diet consisting of medium-chain fatty acid. In R-RPLND group, only one case (7.1%) of low-grade complication, which was an incision infection treated with targeted antibiotics and enhanced dressing change, was recorded. Meanwhile, four episodes (28.6%) of high-grade complications, which was chylous ascites, were observed and treated with the same way as described. In O-RPLND group, there were 2 (28.5%) cases of low-grade complications, including one case of post-operative anemia requiring transfusion and one case of enteritis treated with intravenous infusion, and one case (14.2%) of chylous ascites. Although the rate of low-grade complications was higher and high-grade complication was lower in R-RPLND group than in other groups, the difference was not statistically significant. Of all 47 patients, 13 (27.7%) suffered erectile dysfunction after six months of the surgery, 7 (26.9%) in L-RPLND group, 4 (28.6%) in R-RPLND group, and 2 (28.6%) in O-RPLND group. Only one patient in O-RPLND was not able to ejaculate antegrade during follow-up. The andrological outcomes were comparable. After the surgery, 19 patients attempted to have a child and all of them succeeded. No patients were converted into open surgery in both L-RPLND group and R-RPLND group (Table 3).

## Discussion

This is the first comparative study with three cohorts in one center. Although long-term survival of patients with stage I or II NSGCTs with initial observation, primary RPLND or chemotherapy is pretty optimistic [14], thoroughly conducted surgical treatment still provides more significant therapeutic and staging implications [15]. The potential hazards of chemotherapy also make RPLND a more acceptable option [16]. Meanwhile, studies have confirmed the safety and feasibility of primary RPLND [17, 18]. Therefore, the majority of low stage NSGCT patients in our center received surgery as initial treatment.

After the introduction of R-RPLND in 2006 [19], several comparisons and case series reports have proved its consistency in oncological outcomes with standard O-RPLND [17, 20, 21]. Meanwhile, R-RPLND demonstrated its superiority over O-RPLND in peri-operative outcomes, especially operative duration, blood loss and length of stay [8, 10]. Our study also confirmed the same results as discussed above. Moreover, R-RPLND was able to retrieve more lymph nodes, which is relative with improved prognosis, according to Bhanvadia et al. [22]. We also discovered that the blood tests results of patients in R-RPLND group was less impinged on than those receiving O-RPLND. Specifically, the white blood cell count indicating the level of inflammation was less increased in R-RPLND group. Meanwhile, the level of red blood cell and hemoglobin was higher in R-RPLND group, which might be beneficial for faster post-surgery recovery. These differences were probably due to less surgical injuries and less intra-operative fluid loss.

However, attention should be paid when making therapeutic decisions since controversy about the new technique implies its potential drawbacks. In a series reported by Calaway et al. [7], four patients experienced out-of-field recurrence in unusual sites including sigmoid colon, liver, perinephric space, and lymph nodes in the celiac axis. Potentially explained by poor patient selection, operative technical problems and the inherent defects of surgical technology, such untypical metastasis suggested that the practice of new approach might increase the treatment burden by additional surgeries or chemotherapies. Fortunately, in our study such recurrence did not occur, and the recurrence rates of all three techniques, including standard O-RPLND, were comparable after a relatively long follow-up. It is still debatable whether the adverse outcomes of the small case series are pure coincidences or there is indeed superiority of O-RPLND over R-PRLND in safety.

Lymph leakage is another complication requiring attention. In a Swedish case series report by Bergdahl et al. [23], the incidence of chylous ascites was higher in post-chemotherapy R-RPLND group than O-RPLND group (11% vs. 6.9%). Although primary resections have a lower probability of complication than post-chemotherapy surgeries partly due to desmoplastic reaction induced by cellular toxicity drugs, in our primary surgery cohorts, the same trend of more chylous-related complication in robotic operation group existed (28.6% vs. 14.2% in R- and O-RPLND, respectively). Previous study also confirmed this phenomenon [17], proper interpretations include less suture and clip use during dissection under robotic assisted condition, more lymph nodes retrieved resulting in more lymphatic vessel injuries. Conservative managements such as percutaneous drainage and low-fat diet were enough to solve the problem [24]. Apart from chylous ascites, there were less low-grade complications in R-RPLND group, indicating its advantage in preventing tissue damage.

With the advancement of laparoscopic technology, L-RPLND has become the mainstream operative approach in our center. Experiences from a high-volume center [25] comprehensively elaborated the efficacy of this technique, recommending its substitute for O-RPLND despite of the technical challenges. Other reports, including ours, also supported the superiority of L-RPLND over O-RPLND because of their comparable oncological outcomes and a higher morbidity rate of O-ROLND [26, 27].

Both aiming to provide minimally invasive operation, L-RPLND and R-RPLND are equivalent in terms of feasibility, safety, and perioperative consequences, and oncological outcomes [12]. As a potential alternative to laparoscopic technique, the robot provides better visualization and higher level of freedom of movement without prolonging actual operation time. These advantages contribute to the superior perioperative outcomes in prostatectomy and nephrectomy [28]. However, robotic technique may increase the risk of major bleeding if vascular injury with unsuccessful robotic repair occurred. Meanwhile, under unsterile circumstance, the conversion to open surgery takes more time. Laparoscopic surgery can manage such intraoperative accidence with a Satinsky clamp and a quicker open conversion. Therefore, an experienced bedside assistant is significant in robotic surgery. From our experience, no major vascular injuries were recorded, and the main inferiorities of R-RPLND compared with L-RPLND were longer operative duration due to the set-up and docking of the robotic system, higher chylous leak ratio, and increased hospital expenses.

Apart from oncological and perioperative outcomes, our study also assessed the impacts of these modalities on the andrological functions and fertility capacity of young male adults. Meanwhile, we tested liver and kidney functions by examining pre- and post-operative level of albumin, total bilirubin, and creatinine. Only one patient suffered from retrograde ejaculation after O-RPLND, indicating the advantage of minimally invasive surgery on protecting nerves. Though not statistically significant, lower level of albumin and more increased level of total bilirubin implied the adverse impacts of R-RPLND on liver, partly due to its longer operative time.

Limitations to the current study include the small size, particularly of O-RPLND group, thus limiting its capacity of detecting differences between groups. Moreover, the retrospective design of this study cannot validate the superiority of L-RPLND and R-RPLND. However, this study is still meaningful for providing accessible data for future analysis, particularly when cases of NSGCT are very rare. Further randomized prospective clinical trials are necessary to recommend a standard surgery for NSGCT.

## Conclusion

In the present study, we confirmed the consistency of all three surgical techniques in safety, oncological, andrological, and reproductive outcomes under the circumstance of not using primary chemotherapy. Laparoscopic surgery might be the most cost-effective option and should be recommended as the first-line treatment, but further studies are needed to fully validate the result.

## Data Availability

Raw data were generated at the First Affiliated Hospital, Zhejiang University School of Medicine. Derived data supporting the findings of this study are available from the corresponding author XJ on request.
